# Detection of Small Debonding Defects in Metal–Rubber Bonded Structures Using an Enhanced EMAT and Multi-Feature Fusion Imaging

**DOI:** 10.3390/s26092617

**Published:** 2026-04-23

**Authors:** Yang Fang, Xiaokai Wang, Yinqiang Qu, Hongen Chen, Zhenmao Chen

**Affiliations:** State Key Laboratory for Strength and Vibration of Mechanical Structures, Shaanxi Engineering Research Centre of NDT and Structure Integrity Evaluation, School of Aerospace Engineering, Xi’an Jiaotong University, Xi’an 710049, China; 2321874800@stu.xjtu.edu.cn (X.W.); qyq120140@stu.xjtu.edu.cn (Y.Q.); chenhn@xjtu.edu.cn (H.C.)

**Keywords:** electromagnetic acoustic testing (EMAT), metal-rubber bonded structure, enhanced magnetic field EMAT, multi-feature fusion imaging, quantitative evaluation of debonding area

## Abstract

To improve the low sensitivity of electromagnetic acoustic testing (EMAT) to micro-debonding defects in metal–rubber bonded structures, this study proposes a detection framework combining a magnetic-field-enhanced focusing EMAT with entropy-weighted multi-feature fusion imaging. First, a Halbach-type focusing magnet was designed and evaluated through finite element simulations, showing a substantial enhancement of the effective bias magnetic field in the working region. Then, three complementary echo features, namely amplitude (AMP), time-domain integral (TDI), and power spectral density (PSD), were extracted from the acquired resonance signals and integrated using an adaptive entropy-weighted fusion strategy. Comparative and ablation analyses were further conducted to distinguish the respective contributions of probe enhancement and feature fusion, and to compare entropy-weighted fusion with single-feature imaging and equal-weight fusion. The results indicate that the focused probe mainly improves the defect-response strength at the hardware level, whereas feature fusion mainly improves image contrast, background suppression, and segmentation consistency at the image level. Among the compared methods and under the present experimental conditions, entropy-weighted fusion provides the best overall imaging performance. Under the present experimental conditions, the proposed framework enables reliable detection of 5 mm debonding defects in aluminum-alloy–rubber bonded specimens and 10 mm debonding defects in titanium-alloy–rubber bonded specimens. These results suggest that the combined use of magnetic-field focusing and adaptive multi-feature fusion is a promising approach for the detection and quantitative characterization of micro-debonding defects in metal–rubber bonded structures.

## 1. Introduction

Metal–rubber bonded structures are widely employed in critical engineering applications, especially in aerospace thermal protection and vibration isolation systems [1,2]. Owing to the substantial differences in the physical and mechanical properties of metallic and nonmetallic materials, adhesive bonding has become the most commonly adopted joining method for such structures. However, this joining approach is highly sensitive to processing conditions. For example, inadequate surface treatment or process fluctuations can easily cause debonding defects, thereby compromising the bonding quality. In addition, during service, transportation, and storage, variations in environmental conditions, impact loading, and material curing or settlement may all reduce interfacial bonding strength and even lead to debonding. Such defects can severely impair the durability and reliability of bonded structures, posing potential threats to their service life and operational safety [1,2]. Therefore, nondestructive testing and quantitative characterization of defects, especially interfacial debonding defects, are of great engineering significance. At present, a variety of nondestructive testing techniques have been developed for multilayer heterogeneous structures containing titanium alloys, including infrared thermography [3,4,5], X-ray inspection [6], terahertz imaging [7], and laser speckle detection methods [8,9]. However, for metal–nonmetal multilayer heterogeneous bonded structures, these techniques are often limited by insufficient sensitivity to closed or micro-scale interfacial debonding, strong dependence on operating conditions and surface states, and high implementation costs in field applications.

Electromagnetic acoustic testing (EMAT), as a non-contact ultrasonic inspection technique, has shown considerable potential in the inspection of metallic and multilayer structures. Electromagnetic acoustic transducers (EMATs) can excite and receive ultrasonic waves without the need for a couplant, which gives them unique advantages in scenarios involving high temperatures, complex interfaces, or restricted surface conditions. Extensive studies have been conducted worldwide on EMAT technology, including transduction mechanisms and multiphysics coupling modeling, probe structure design and parameter optimization, and signal processing, imaging, and quantitative characterization methods, thereby laying the foundation for its application to defect detection in complex structures. A thorough understanding and accurate modeling of the EMAT transduction mechanism are essential for improving excitation efficiency and defect detectability. Kawashima et al. [10] systematically elucidated the mechanism of acoustic field generation by EMATs in non-ferromagnetic materials and revealed the excitation processes of bulk waves and surface waves. Sun et al. [11] systematically reviewed numerical, analytical, and semi-analytical modeling methods and analyzed their respective advantages and limitations. Ma et al. [12] proposed an improved non-contact EMAT method for debonding detection in metal-based composite structures using finite element simulation, in which a carbonyl iron powder backing plate was introduced beneath a small butterfly coil to enhance interfacial detection capability. Qi et al. [13] focused on the influence of coil structure design on signal characteristics, providing guidance for EMAT structural optimization. In practical applications, EMAT inspection often suffers from low signal-to-noise ratio (SNR), waveform distortion, and electromagnetic interference, which necessitates the use of advanced signal processing techniques to improve detection performance. To this end, a variety of signal enhancement methods have been proposed, such as wavelet threshold denoising [14], model-guided singular value decomposition (SVD) [15], and variational mode decomposition combined with adaptive interpolation [16]. From the perspective of engineering application, substantial progress has also been made in EMAT system design. Tu et al. [17] proposed a circular-array EMAT that employed a single coil and multiple permanent magnets to achieve full-cross-section inspection of steel pipes. Nakamura et al. [18,19] designed a point-focusing EMAT that realized the detection of stress corrosion cracks in stainless steel through phase-focused superposition of shear waves. Pei et al. [20] developed a flexible robotic scanning system that, combined with resonance signals and power spectral density (PSD) analysis, effectively improved the SNR for debonding detection in metal–rubber structures. In addition, Song et al. [21,22] established a probability of detection (POD) model considering factors such as groove depth and surface roughness, and validated it experimentally using 41 carbon steel plate specimens with artificial corrosion grooves.

Despite these advances, significant challenges remain in the detection and imaging of micro-debonding defects in metal–rubber bonded structures, particularly in terms of low transduction efficiency, weak defect echo signals, and reduced detection capability in low-conductivity substrates such as titanium alloys. Therefore, improving EMAT transduction efficiency, optimizing magnetic field structure design, and developing high-resolution signal processing and imaging methods remain key research priorities.

To address these challenges, this study proposes a micro-debonding detection framework for metal–rubber bonded structures based on Electromagnetic Ultrasonic Resonance (EMUR), by combining a magnetic-field-enhanced focusing EMAT with entropy-weighted multi-feature fusion imaging. First, the response characteristics of interfacial echo signals under different bonding conditions are analyzed, and the Lorentz-force-based shear-wave excitation mechanism is examined. On this basis, a Halbach-type focusing magnet is designed to enhance the effective bias magnetic field in the working region and thereby improve the defect-response strength. Subsequently, an automated planar-scanning inspection system is established to acquire experimental data from aluminum-alloy–rubber and titanium-alloy–rubber specimens containing debonding defects of different sizes. Based on the acquired resonance signals, three complementary features, namely amplitude (AMP), time-domain integral (TDI), and power spectral density (PSD), are extracted and integrated using an adaptive entropy-weighted fusion strategy. The proposed framework is finally used to achieve imaging and quantitative evaluation of micro-debonding defects in two representative metal–rubber bonded systems.

## 2. Methodology

### 2.1. EMAT Detection Mechanism for Metal–Rubber Debonding

**Figure 1:**
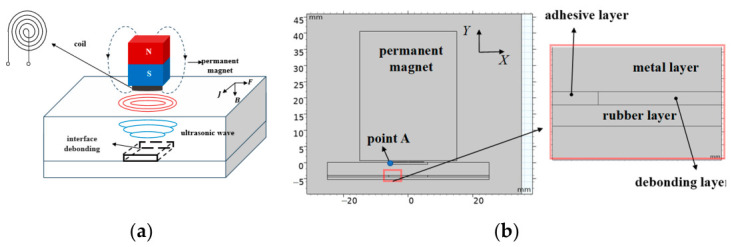
Schematic of the EMAT model: (**a**) operating principle; (**b**) COMSOL model for electromagnetic ultrasonic inspection with defects.

To investigate the response characteristics of Electromagnetic Ultrasonic Resonance (EMUR) signals in metal–nonmetal bonded structures and their sensitivity to interfacial debonding defects, numerical simulations were first conducted. A two-dimensional EMAT simulation model was established for detecting debonding defects at the metal–rubber bonding interface, as shown in Figure 1. A typical electromagnetic acoustic transducer (EMAT) consists of a permanent magnet, an excitation/receiving coil, and a backing structure, and enables ultrasonic wave excitation and reception without the need for coupling media or mechanical contact. For metal–rubber bonded structures, ultrasonic echo responses are primarily governed by the mechanical coupling condition at the interface. When the interface is well bonded, ultrasonic energy can be partially transmitted into the rubber layer and dissipated, resulting in relatively smooth and attenuated echo characteristics. In contrast, when interfacial debonding occurs, the local acoustic impedance mismatch increases significantly, leading to enhanced reflection and potentially stronger localized resonance responses within the metal layer. This phenomenon provides a physical basis for the detection and characterization of debonding defects. It should be noted that, since rubber is an electrically insulating material, EMAT energy input occurs predominantly within the metal layer. Therefore, defect information is indirectly reflected through the interaction between the ultrasonic field in the metal layer and the altered interfacial boundary conditions. This indirect sensing mechanism imposes higher requirements on excitation efficiency and SNR for reliable defect detection.

The primary transduction mechanisms of EMAT in conductive materials include the Lorentz-force effect and the magnetostrictive effect. For non-ferromagnetic or weakly ferromagnetic metals, such as aluminum alloys and titanium alloys, the contribution of magnetostriction is typically negligible, and the transduction process is dominated by the Lorentz-force mechanism. When an alternating current is applied to the excitation coil, an alternating electromagnetic field is generated at the metal surface, inducing eddy currents J within the conductive material. Under the presence of a static bias magnetic field B_0_, these eddy currents interact with the magnetic field to produce a volumetric body force, which serves as the ultrasonic excitation source. The corresponding equivalent body force density can be expressed as:

(1) $$f_{L}=J\times B_{0}$$

As indicated by the above equation, under a given coil-current condition, the ultrasonic excitation strength is directly proportional to the product of $B_{0}$ and J. For weakly ferromagnetic metals or materials exhibiting magnetoelastic coupling, magnetostrictive effects may also contribute; the corresponding equivalent driving force depends on the magnetization state and can generally be summarized as: the more fully the material is magnetized and the more appropriately the bias magnetic field is applied, the stronger the magnetostrictive effect. Therefore, regardless of whether the dominant mechanism is the Lorentz force or magnetostriction, increasing the effective bias magnetic field strength and optimizing its spatial distribution are key approaches to improving EMAT transduction efficiency and the SNR of the received echoes.

In addition, the eddy-current distribution is governed by the material electrical conductivity σ, magnetic permeability μ, and the excitation angular frequency ω. A commonly used characteristic parameter is the electromagnetic skin depth δ:

(2) $$\delta =\sqrt{\frac{2}{\omega \mu \sigma }}$$

This parameter determines the effective penetration (interaction) thickness of the induced current and its degree of spatial confinement, thereby affecting both the magnitude and spatial profile of J. Because aluminum and titanium alloys differ markedly in electrical conductivity and magnetic properties, their eddy-current intensity and skin depth are different under the same excitation frequency and coil configuration. As a result, the strength and spatial extent of the Lorentz-force source term $J\times B_{0}$ vary accordingly, which manifests as differences in EMAT excitation efficiency, echo amplitude, and sensitivity to noise. Therefore, for relatively low-conductivity materials such as titanium alloys, improving the strength and focusing capability of the bias magnetic field is particularly important for enhancing detection sensitivity.

Conventional shear-wave EMATs typically employ a single permanent magnet. However, the vertical magnetic field component within the effective coil interaction region, especially in the central area, is usually weak. This results in a reduced Lorentz-force driving term, leading to lower ultrasonic echo amplitude and insufficient SNR. The problem becomes more severe in the detection of small-scale debonding defects, whose weak echoes are easily masked by noise.

### 2.2. Design of a Magnetic-Field-Enhanced EMAT

**Figure 2:**
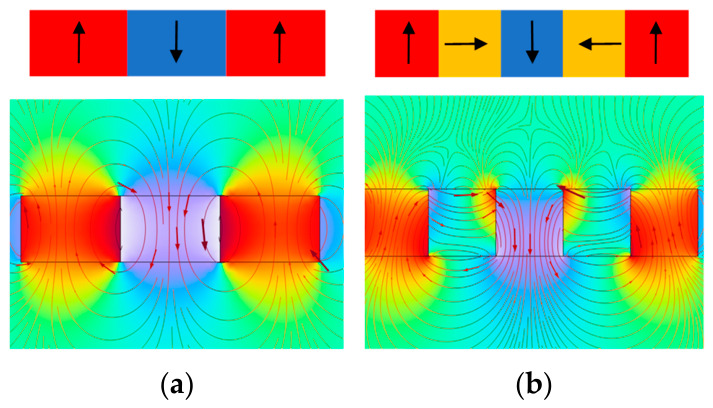
Magnetization directions of different magnetic configurations: (**a**) conventional permanent-magnet arrangement; (**b**) Halbach permanent-magnet array.

To overcome the insufficient vertical bias magnetic field in conventional EMAT magnetic circuits, the magnetic-field enhancement principle of the Halbach permanent-magnet array was introduced. Compared with the conventional magnet configuration, the Halbach array concentrates magnetic flux on the working side by successively rotating the magnetization directions of adjacent magnets, thereby enhancing the intensity and uniformity of the vertical bias magnetic field. Figure 2a,b show the magnetization directions of the conventional magnet configuration and the conventional Halbach array, respectively. Inspired by this mechanism, a ring-shaped Halbach array was designed in this work to better match the circular spiral coil.

**Figure 3:**
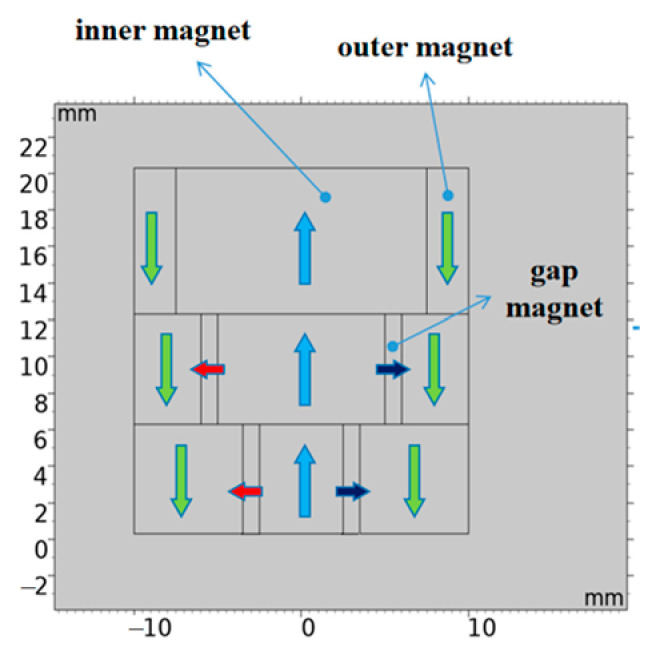
Ring-type Halbach array focusing magnet.

To satisfy the geometric compatibility requirements of the circular spiral excitation coil used in the experiments, while maintaining structural symmetry, magnetic field focusing capability, and installation compatibility, the proposed ring-shaped Halbach focusing magnet configuration is shown in Figure 3 (The arrows in the picture indicate the direction of the magnet.). The magnet structure consists of three layers:

(1) Inner layer: an axially magnetized cylindrical magnet;
(2) Outer layer: an axially magnetized annular magnet;
(3) Middle layer: a set of sector magnets with magnetization directions oriented radially outward in the central region.

This magnetization arrangement produces a stronger flux-concentration effect on the working side, significantly enhancing the magnetic field within the central effective region. As a result, it provides more favorable bias-field conditions for high-efficiency shear-wave excitation.

**Figure 4:**
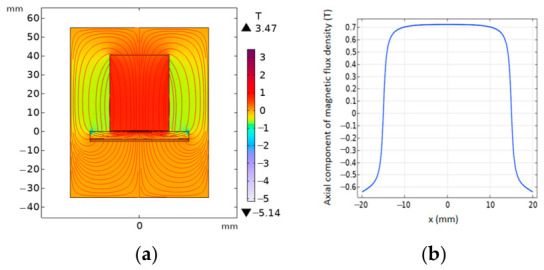
Magnetic field distribution: (**a**) distribution of the magnetic field; (**b**) magnetic flux density distribution.

**Figure 5:**
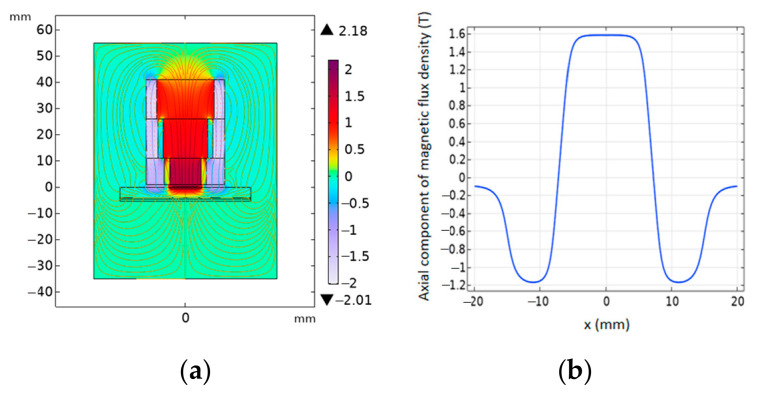
Magnetic field distribution: (**a**) ring-shaped Halbach focusing magnet; (**b**) magnetic flux density distribution.

**Figure 6:**
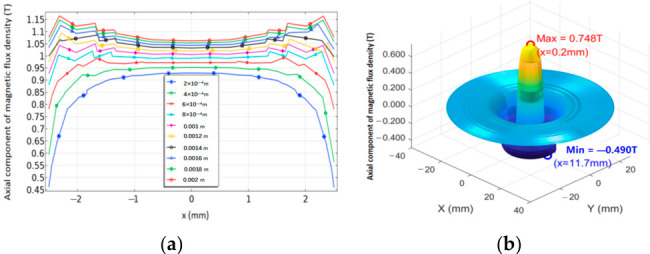
Magnetic flux density distribution: (**a**) effect of the spacing magnets; (**b**) three-dimensional mapping of magnetic flux density.

To quantitatively evaluate the magnetic field enhancement effect of the proposed magnet configuration, three-dimensional finite element models of both the conventional EMAT and the ring-shaped Halbach EMAT were established using COMSOL Multiphysics^®^ 6.2. The models include the permanent magnet structures, surrounding air domain, and an observation plane, with the magnetostatic physics module employed to solve for the spatial distribution of the magnetic flux density $B_{0}$. To correspond with the actual working region of the excitation coil, a plane located 1 mm above the magnet surface was selected as the evaluation cross-section. Particular attention was given to the vertical component of the magnetic flux density, $B_{y}$ (normal to the specimen surface), which is directly related to shear-wave excitation efficiency (Figure 4, Figure 5 and Figure 6). To further investigate the influence of the Halbach array parameters on magnetic field focusing performance, a parametric analysis was conducted on the geometric dimensions of the intermediate fan-shaped magnets (spacing magnets). By comparing the peak magnitude and spatial concentration characteristics of the vertical magnetic flux density in the central region under different parameter configurations, the contribution of these parameters to magnetic field enhancement was evaluated, and the corresponding optimization strategy was identified.

Simulation results show that, on the observation plane located 1 mm above the magnet surface, the annular Halbach focusing magnet produces a pronounced flux-concentration effect in the central region. Specifically, the central field strength increases from 0.70 T for the conventional configuration to 1.60 T in this study, corresponding to a 2.29-fold improvement. Meanwhile, the magnetic-field focusing width decreases from 25.5 mm to 9.7 mm, representing a 62% reduction, indicating a more concentrated symmetric field distribution. In contrast, the conventional cylindrical magnet exhibits a lower $B_{y}$ magnitude in the central region and a more evident field spreading. As the size of the intermediate-layer sector magnets increases, the central peak value of $B_{y}$ further rises and reaches its maximum within the effective working region, suggesting that this geometric parameter plays a critical role in determining the focusing capability of the magnetic circuit.

Because the magnetic field is more concentrated and uniform in the central effective region, the proposed magnet configuration facilitates a more stable acoustic-field distribution and mitigates in-plane excitation non-uniformity. This trend is also reflected in the simulated ultrasonic displacement fields: compared with the conventional magnet, the Halbach focusing magnet generates an in-plane displacement distribution that is more uniform, which is expected to yield more consistent scanning and imaging performance in practical inspections.

### 2.3. Imaging Based on Entropy-Weighted Fusion

To obtain a stable ultrasonic resonance response within the debonded region, the excitation frequency of the long tone-burst is set according to the standing-wave condition of shear waves along the thickness direction of the upper metal plate:

(3) $$f=\frac{nc}{2d}$$

where f denotes the EMAT excitation frequency, c is the shear-wave velocity in the upper layer, d is the thickness of the upper structure, and n is a positive integer representing the resonance mode order. When the EMAT is located above a debonded region, the top and bottom surfaces of the upper metal plate can be approximately treated as traction-free boundaries, which facilitates the formation of standing-wave resonance along the thickness direction and results in a pronounced increase in echo energy. In contrast, in well-bonded regions, part of the ultrasonic energy is coupled through the adhesive layer into the lower structure, suppressing the resonance effect; consequently, distinguishable differences arise in frequency-domain and energy-related metrics.

Let the resonance signal acquired at a given measurement point be denoted as s(t). Within a selected analysis time window $\left[t_{1},\;t_{2}\right]$, three types of features were constructed and used for two-dimensional imaging.

(1) Amplitude feature

This feature captures the intuitive variation in echo strength. In this study, the peak-to-peak amplitude was used.

(4) $$\mathrm{AMP}=\max_{t\in [t_{1},t_{2}]}s(t)$$

This feature is sensitive to strongly reflecting defects; however, under low-SNR conditions it is susceptible to random noise and impulsive spike interference.

**Figure 7:**
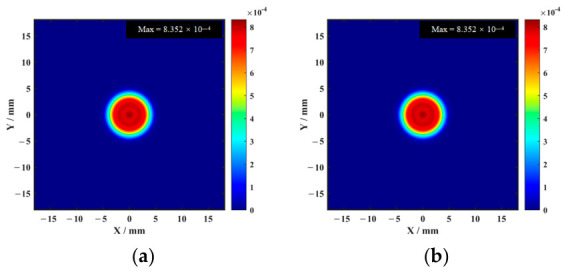
Amplitude imaging simulation results: (**a**) enhanced magnet; (**b**) conventional magnet.

Amplitude imaging simulation results are shown in Figure 7.

(2) Time-domain integral feature

Given that resonance signals typically exhibit long durations and pronounced energy accumulation, time-domain energy- or envelope-based integral metrics can improve robustness. In this study, a commonly used form (energy integral) is adopted:

(5) $$\mathrm{TDI}=\int_{t_{1}}^{t_{2}}s^{2}(t)dt$$

**Figure 8:**
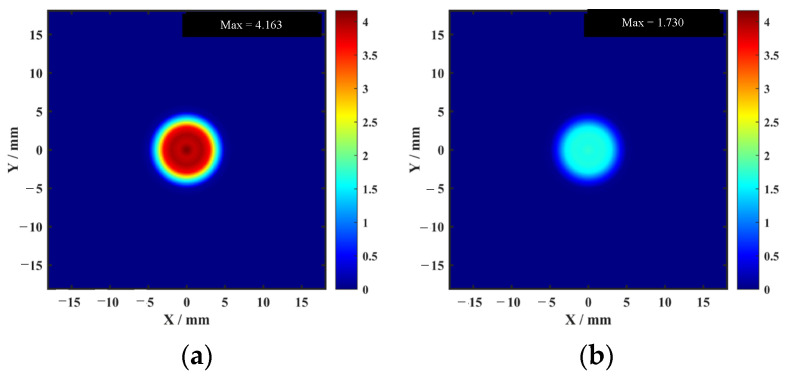
Time-domain integral imaging simulation results: (**a**) enhanced magnet; (**b**) conventional magnet.

As shown in Figure 8, compared with the single-point amplitude, the integral feature suppresses the influence of sporadic noise and is more robust for weak defects.

(3) Power spectral density (PSD) feature

To better exploit the frequency-domain energy concentration of resonance signals, a PSD-based feature was introduced. The resonance signal was first analyzed via autocorrelation:

(6) $$R(m)=\sum_{n}s(n)s(n+m)$$

where R(m) denotes the autocorrelation function, s(n) denotes the discrete signal, and m is the lag. Because the autocorrelation of random noise tends to zero as the lag increases, autocorrelation analysis can effectively suppress random noise and emphasize periodic components.

The PSD is then defined based on the autocorrelation–Fourier transform relationship, with a window function W(m) applied:

(7) $$P(f)=\Delta t\sum_{m=-M}^{M}W(m)R(m)e^{-j2\pi fm\Delta t}$$

where P(f) is the power spectral density at frequency f, W(m) is the window function, and Δt is the sampling interval. Unlike the discrete Fourier transform (DFT), which primarily reflects spectral-line amplitude and phase, the PSD characterizes how signal power is distributed over frequency and is therefore more suitable for describing the degree of resonance-energy concentration around the target frequency. Since a debonded region is expected to produce a stronger standing-wave resonance response, its spectral energy typically forms a pronounced peak near the resonance frequency. Accordingly, the PSD peak value is adopted as the third feature in this study:

(8) $$\mathrm{PSD}=\max_{f\in [f_{a},f_{b}]}P(f)$$

where $\left[f_{a},\;f_{b}\right]$ denotes the analysis bandwidth selected around the resonance frequency f. This peak feature is more sensitive to resonance enhancement and is generally more robust than time-domain amplitude under low-SNR conditions, which is beneficial for improving the detectability of small debonding defects.

**Figure 9:**
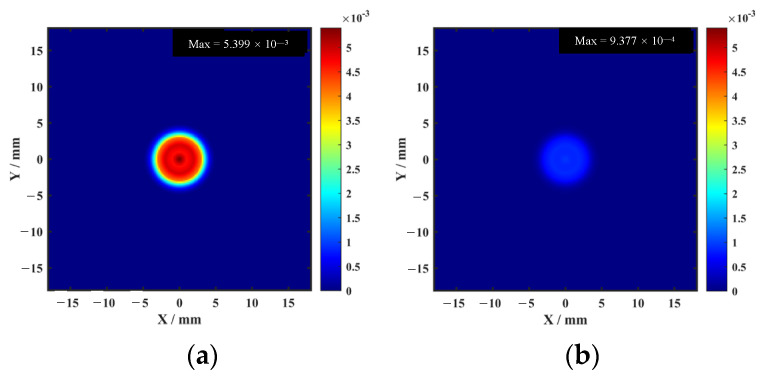
PSD-based imaging simulation results: (**a**) enhanced magnet; (**b**) conventional magnet—10 mm delamination.

PSD-based imaging obtained using simulated data, as shown in Figure 9.

Accordingly, three feature maps can be obtained for each scan point, i.e., AMP(x,y), TDI(x,y), PSD(x,y). Because these features differ in defect sensitivity and noise-suppression capability, imaging based on a single feature alone often cannot simultaneously achieve high contrast and robustness; therefore, multi-feature fusion is further required.

To avoid the subjectivity associated with manually assigned weights and to enable an adaptive evaluation of the information content of different features, entropy weighting was adopted for feature fusion. Let the i-th feature map be denoted as P(f) (corresponding to AMP, TDI, and PSD, respectively). Each feature map is first converted to a nonnegative form and normalized, and is then treated as a two-dimensional probability distribution.

(9) $$p_{i}(x,y)=\frac{F_{i}(x,y)}{\sum_{x,y}F_{i}(x,y)+\varepsilon }$$

where ε is a small constant introduced to prevent division by zero. Subsequently, the information entropy of the i-th feature is calculated as:

(10) $$H_{i}=-\sum_{x,y}p_{i}(x,y)\ln(p_{i}(x,y)+\varepsilon )$$

The entropy value reflects the distribution uniformity of the feature. A larger entropy indicates a more uniform distribution and weaker discriminative capability, whereas a smaller entropy indicates that the energy is more concentrated and that the feature contains more effective information. The information utility value is therefore defined as:

(11) $$d_{i}=1-H_{i}$$

Based on this, the adaptive fusion weight for each feature can be obtained as:

(12) $$w_{i}=\frac{d_{i}}{\sum_{j}d_{j}}$$

The final fused image is generated by the weighted superposition of the three feature maps followed by normalization:

(13) $$\mathrm{Fusion}(x,y)=\mathrm{norm}\left(\sum_{i=1}^{3}w_{i}F_{i}(x,y)\right)$$

where $\mathrm{norm}\left(•\right)$ denotes the normalization operation, which facilitates subsequent threshold segmentation and geometric quantification. Through the entropy weight method, the weights can adaptively adjust according to the data characteristics. Specifically, when a feature map exhibits a more concentrated energy distribution in the defect region (corresponding to lower information entropy), its weight is automatically increased, thereby playing a dominant role in the fused result. Conversely, when a feature is affected by noise and its distribution becomes more uniform, its weight is automatically reduced, which helps improve the robustness and reliability of the final imaging result.

### 2.4. Quantitative Assessment of Defects

#### 2.4.1. Otsu-Based Defect Segmentation

To enable quantitative characterization of debonding defects, the fused image was further processed using automatic threshold-based segmentation. Since the fused image enhances the contrast between defective and intact regions, its grayscale histogram provides an effective basis for threshold determination. In this study, Otsu’s maximum between-class variance method was adopted to automatically determine the optimal segmentation threshold, owing to its simplicity, reproducibility, and suitability for images with distinguishable foreground–background gray-level distributions.

Let the candidate threshold be denoted by T. The fused image is then divided into two classes: the defect region A and the non-defect (background) region B. For a given threshold T, the between-class variance is defined as:

(14) $$\sigma_{b}^{2}(T)=\omega_{0}(T)\omega_{1}(T){\left[\mu_{0}(T)-\mu_{1}(T)\right]}^{2}$$

where $\omega_{0}(T)$ and $\omega_{1}(T)$ represent the proportions of pixels belonging to the background class and the target class, respectively, $\mu_{0}(T)$ and $\mu_{1}(T)$ denote the corresponding mean gray levels. A larger between-class variance indicates stronger separability between the two classes and, consequently, a better segmentation effect. Therefore, the optimal threshold T is determined by:

(15) $$T^{*}=\arg\max_{T}\sigma_{b}^{2}(T)$$

Once the optimal threshold is obtained, the fused image F(x,y) is binarized as:

(16) $$B(x,y)=\left\{\begin{array}{c} 1,\;F(x,y)\geq T^{*}\\ 0,\;F(x,y)<T^{*} \end{array}\right.$$

where B(x,y) denotes the binary segmentation result. Pixels with gray levels greater than or equal to $T^{*}$ are classified as belonging to the defect region, whereas those with gray levels lower than $T^{*}$ are treated as background. Through this process, the high-response debonding region can be separated from the low-response background, yielding a clear binary defect map for subsequent quantitative analysis.

Based on the segmented binary image, the defect contour and geometric parameters were extracted for quantitative evaluation. Specifically, the segmented defect area was calculated from the number of foreground pixels, and the corresponding equivalent diameter was estimated by converting the segmented area into that of an equivalent circle.

#### 2.4.2. Quantitative Metrics

(1) Signal-to-noise ratio (SNR)

The SNR was used to evaluate the prominence of the defect response relative to the background noise. Following the root-mean-square definition, the SNR is expressed as:

(17) $$SNR(dB)=20\log_{10}\left(\frac{\sqrt{\frac{1}{N_{s}}\sum_{i=1}^{N_{s}}s_{i}^{2}}}{\sqrt{\frac{1}{N_{n}}\sum_{j=1}^{N_{n}}e_{j}^{2}}}\right)$$

where $s_{i}$ denotes the signal samples in the defect region, $e_{i}$ denotes the background-noise samples, $N_{s}$ and $N_{n}$ denote the numbers of signal and noise samples, respectively.

(2) Contrast

To quantify the grayscale separability between the defect region and the background region in the imaging results, the contrast was defined as:

(18) $$C=\frac{\left|\mu_{d}-\mu_{b}\right|}{\mu_{d}+\mu_{b}}$$

where $\mu_{d}$ and $\mu_{b}$ represent the mean grayscale values of the defect region and the background region, respectively.

## 3. Experimental Verification and Analysis

### 3.1. Experimental System

**Figure 10:**
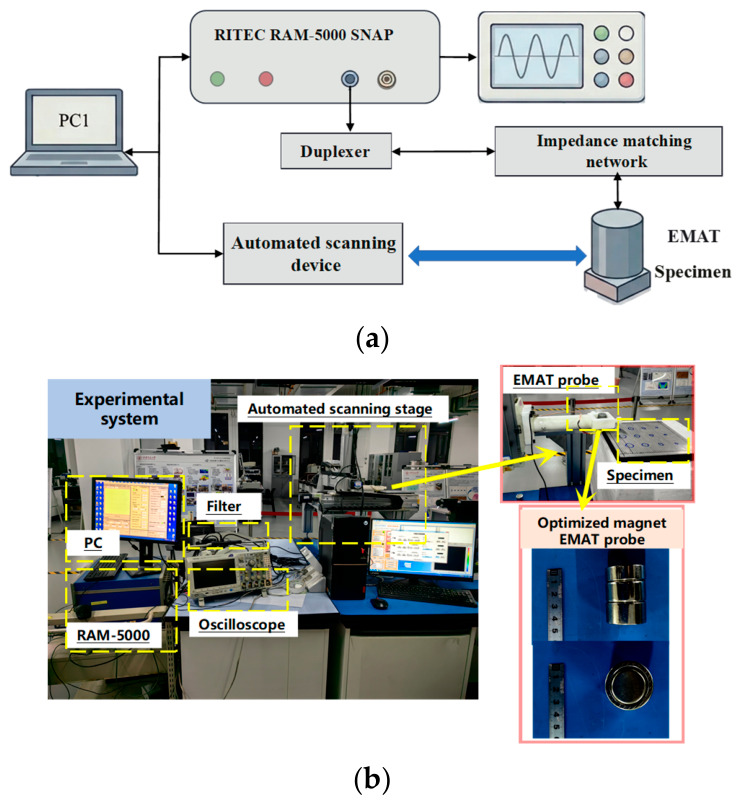
EMUR-based experimental system for planar-scanning NDT of metal–nonmetal interfacial debonding defects: (**a**) schematic illustration; (**b**) picture of the practical system.

To validate the simulation results and to evaluate the feasibility of Electromagnetic Ultrasonic Resonance (EMUR) for detecting debonding at metal–nonmetal interfaces, a two-dimensional planar-scanning NDT system based on the enhanced EMAT was established (Figure 10). The system consists of a computer, a band-pass filter, a RITEC RAM-5000 SNAP unit, an impedance-matching network, an automated scanning stage, a duplexer (Tx/Rx isolation), a preamplifier, an oscilloscope/high-speed data acquisition card, and the bonded specimen under test. The RITEC RAM-5000 SNAP, serving as a high-energy pulser/receiver, generates a narrowband tone-burst excitation signal, which is delivered through the matching network and the duplexer to drive the EMAT for ultrasonic generation. The returning echoes induce a voltage signal in the receive coil; after preamplification and band-pass filtering, the signal is acquired by the oscilloscope and transmitted to the host computer for storage and further processing. The key parameters of the experimental system are summarized in Table 1.

**Figure 11:**
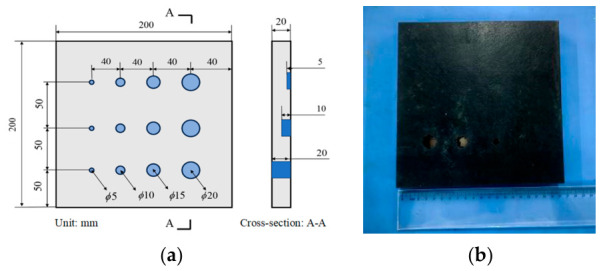
Metal–rubber plate specimen: (**a**) schematic design; (**b**) photograph of the fabricated specimen.

Considering the structural parameters of metal–rubber bonded components under practical service conditions, as well as the nondestructive testing requirements for identifying debonding defect sizes, representative experimental specimens were designed and fabricated in this study. Each specimen (aluminum-alloy–rubber and titanium-alloy–rubber) consisted of a metal plate with dimensions of 200 mm × 200 mm × 4 mm bonded to a rubber plate of 200 mm × 200 mm × 20 mm. To simulate interfacial debonding, circular non-bonded regions of different sizes were introduced at the rubber–metal interface. The debonding diameters were set to 5, 10, 15, and 20 mm. For each diameter, three debonding depths (5, 10, and 20 mm) were further designed, resulting in a total of 12 debonding locations in each specimen. A schematic of the defect layout in the rubber layer is shown in Figure 11a, and a photograph of the fabricated metal–rubber bonded specimen is presented in Figure 11b.

**Figure 12:**
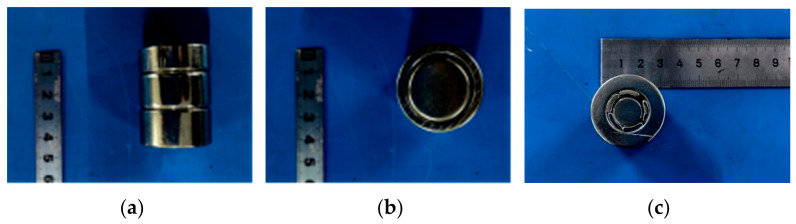
Photograph of the proposed three-layer nested focusing magnet: (**a**) front view; (**b**) top view; (**c**) bottom view.

To enhance the vertical bias magnetic field for shear-wave excitation in the EMAT and to improve magnetic-flux focusing in the central region, the experiments employed the enhanced magnet designed in the previous section. As shown in Figure 12, the focusing magnet comprises three components and the key geometric dimensions are provided in Table 2.

Data acquisition was performed using a two-dimensional planar C-scan. The EMAT probe was translated point-by-point along a raster path on the specimen surface with a step size of 0.1 mm, covering an area of 180 mm × 180 mm. At each position, 16 measurements were acquired and averaged to suppress random noise. To ensure consistent comparison, the probe orientation and lift-off distance were kept constant throughout the scan, maintaining identical incidence conditions. For each measurement point, the time-domain echo s(t) was recorded, and amplitude-related features were extracted to form a 2D amplitude map for conventional imaging.

### 3.2. Experimental Results

#### 3.2.1. Experimental Signal Comparison

**Figure 13:**
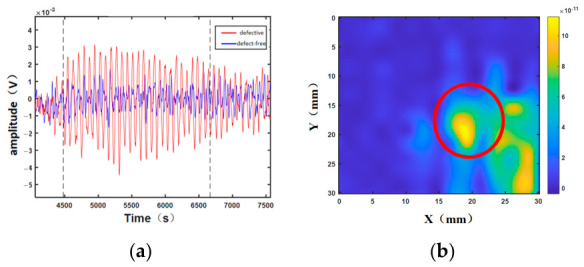
Experimental signals (titanium-alloy–rubber): (**a**) raw signal; (**b**) localized imaging analysis.

Under identical incidence conditions, a comparison of echo signals acquired at the debonding edge and in intact regions indicates that air-layer debonding produces a strong shear-wave reflection, leading to echo amplitudes in the defective area that are significantly higher than those in the well-bonded region (Figure 13). The area marked by the red circle in Figure 13b is the defective area. This observation is consistent with the simulation results, confirming that debonding at the titanium-alloy–rubber interface can be effectively detected via echo variations. However, conventional amplitude-based imaging still suffers from insufficient contrast, blurred boundaries, and interference from background artifacts for certain defects, particularly small-sized or weak debonding.

#### 3.2.2. Imaging and Defect Quantification

**Figure 14:**
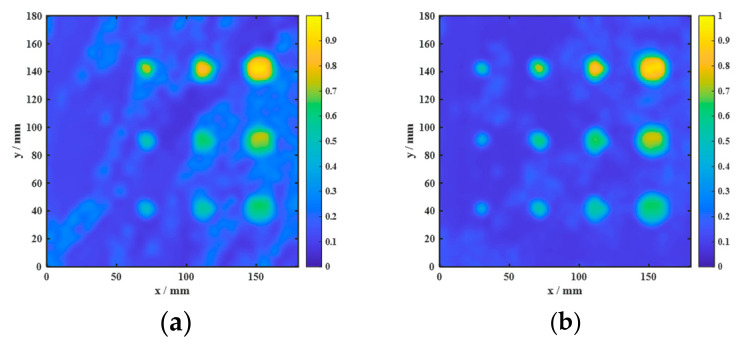
Experimental detection results of aluminum-alloy–rubber debonding defects: (**a**) conventional EMAT probe; (**b**) focused EMAT probe proposed in this study.

**Figure 15:**
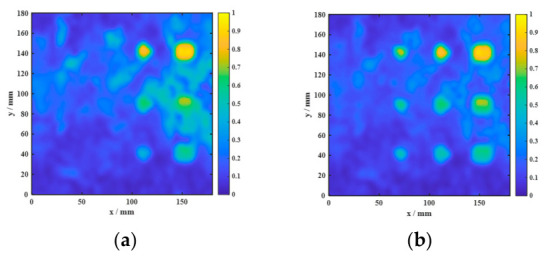
Experimental detection results of titanium-alloy–rubber debonding defects: (**a**) conventional EMAT probe; (**b**) focused EMAT probe proposed in this study.

The data acquired from the aluminum-alloy–rubber and titanium-alloy–rubber specimens were imaged using the proposed imaging method. The results in Figure 14 and Figure 15 show that the combined use of the focused probe and entropy-weighted fusion yields the best overall imaging quality among the compared configurations. For the aluminum-alloy–rubber bonded structure, the focused probe can stably identify a 5 mm diameter debonding defect, exhibiting superior detection capability compared with the conventional probe. For the titanium-alloy–rubber bonded structure, the focused probe benefits from magnetic-field enhancement to achieve a higher SNR and can reliably detect a 10 mm diameter debonding defect.

**Figure 16:**
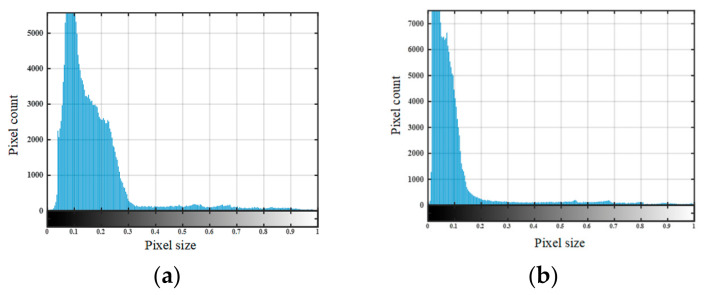
Grayscale histograms of aluminum-alloy–rubber debonding experimental images: (**a**) conventional EMAT probe; (**b**) focused EMAT probe.

**Figure 17:**
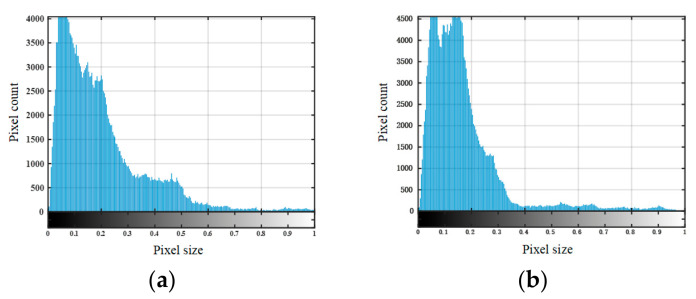
Grayscale histograms of titanium-alloy–rubber debonding experimental images: (**a**) conventional EMAT probe; (**b**) focused EMAT probe.

**Figure 18:**
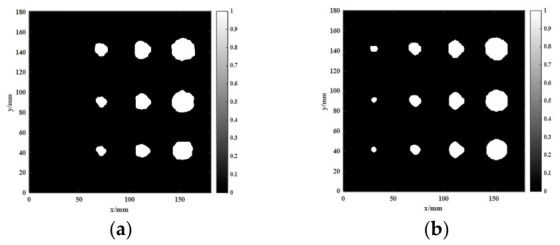
Image segmentation results of aluminum-alloy–rubber debonding defects: (**a**) conventional EMAT probe; (**b**) focused EMAT probe.

**Figure 19:**
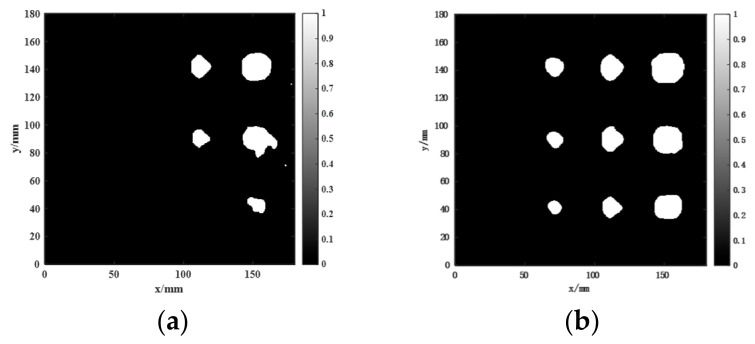
Image segmentation results of titanium-alloy–rubber debonding defects: (**a**) conventional EMAT probe; (**b**) focused EMAT probe.

The two-dimensional imaging results shown in Figure 14 and Figure 15 were quantitatively characterized using the defect quantification algorithm proposed in this study. First, the two-dimensional images were binarized to obtain the corresponding grayscale histograms, as presented in Figure 16 and Figure 17, respectively. Based on these distributions, the optimal segmentation thresholds were determined by maximizing the interclass variance between the target and the background, and the resulting defect segmentation results are shown in Figure 18 and Figure 19, respectively. In this study, for the aluminum-alloy–rubber structure, the thresholds corresponding to the conventional EMAT probe and the focusing EMAT probe were 0.382 and 0.320, respectively. For the titanium-alloy–rubber structure, the corresponding thresholds were 0.375 and 0.384, respectively. Otsu adaptive thresholding applied to the entropy-weighted fusion results provides a practical and reproducible basis for automatic defect extraction under the present experimental conditions for both the aluminum-alloy–rubber and titanium-alloy–rubber bonded structures, providing a consistent basis for subsequent quantitative evaluation in this study of defect geometric parameters. As shown in Figure 18 and Figure 19, compared with the conventional probe, the proposed focused EMAT probe yields binary segmentation results with clearer defect boundaries and higher contrast.

Table 3 and Table 4 summarize the quantitative evaluation results obtained for the aluminum-alloy–rubber structure using the conventional probe and the proposed focused probe, respectively, whereas Table 5 and Table 6 present the corresponding results for the titanium-alloy–rubber structure. For the aluminum-alloy–rubber specimen, comparison of Table 3 and Table 4 shows that the conventional probe fails to effectively detect the defect with a diameter of 5 mm, whereas the proposed probe and method enable successful detection and provide higher quantitative accuracy. Similarly, for the titanium-alloy–rubber specimen, comparison of Table 5 and Table 6 indicates that the conventional probe cannot effectively identify the defect with a diameter of 10 mm, while the proposed probe and method achieve reliable detection with improved quantification accuracy.

In summary, the proposed multi-feature fusion imaging method, combined with Otsu segmentation and implemented using the magnetic-field-enhanced focused EMAT probe, not only improves the detectability of debonding defects but also significantly enhances the accuracy of quantitative defect-area evaluation. This approach provides an effective means for the reliable characterization of debonding damage in metal–rubber multilayer bonded structures.

### 3.3. Experimental Analysis

#### 3.3.1. Performance Analysis of Enhanced Probes and Fusion Algorithms

To clarify the respective contributions of probe-level magnetic-field enhancement and image-level feature fusion, an ablation study was conducted using four configurations: (i) a conventional EMAT probe without feature fusion, (ii) a focused EMAT probe without feature fusion, (iii) a conventional EMAT probe with feature fusion, and (iv) a focused EMAT probe with feature fusion. Here, without fusion condition refers to imaging based on the amplitude feature alone, while the with fusion condition refers to the entropy-weighted fusion of AMP, TDI, and PSD features.

**Figure 20:**
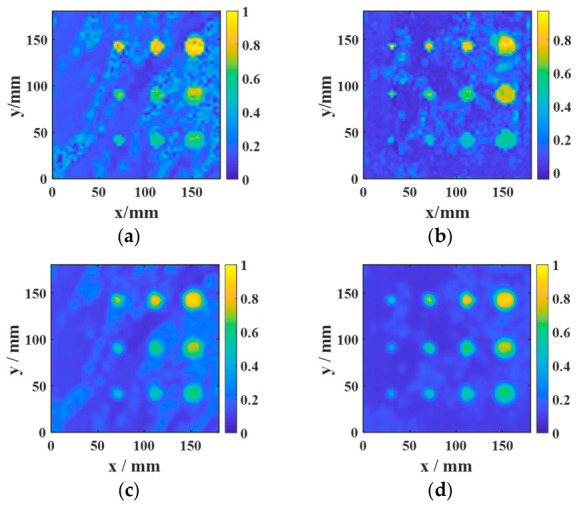
Ablation results for four configurations in the aluminum-alloy–rubber specimen: (**a**) conventional probe without fusion; (**b**) focused probe without fusion; (**c**) conventional probe with fusion; (**d**) focused probe with fusion.

**Figure 21:**
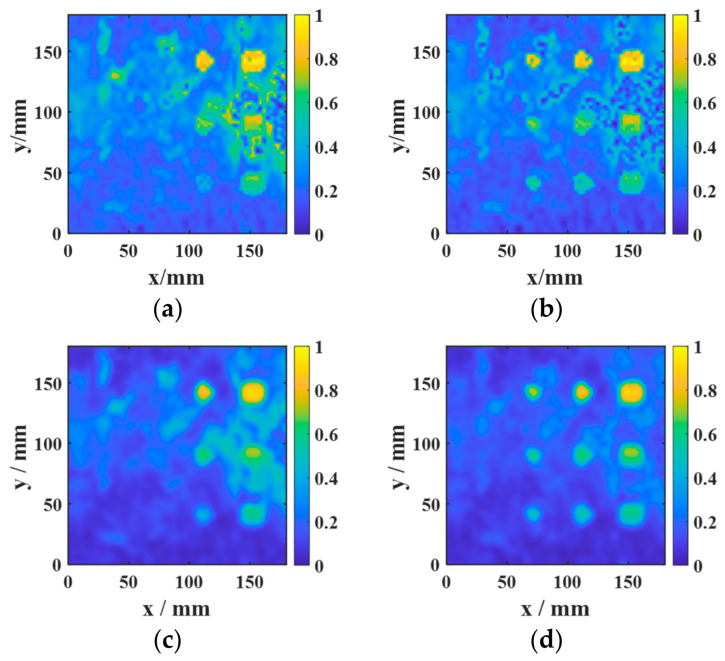
Ablation results for four configurations in the titanium-alloy–rubber specimen: (**a**) conventional probe without fusion; (**b**) focused probe without fusion; (**c**) conventional probe with fusion; (**d**) focused probe with fusion.

Figure 20 and Figure 21 present the ablation results for four configurations in the aluminum-alloy–rubber and titanium-alloy–rubber specimens, respectively. A clear trend can be observed in both material systems. First, under the same image-processing condition, the focused probe consistently provides stronger defect responses than the conventional probe. Specifically, comparing Figure 20a with Figure 20b, and Figure 21a with Figure 21b, the focused probe without fusion already exhibits improved defect visibility and reduced background interference, indicating that magnetic-field enhancement improves signal generation and reception at the hardware level. However, in the absence of feature fusion, small defects still suffer from blurred boundaries and incomplete contours, and this limitation is more evident in the titanium-alloy–rubber specimen due to its relatively weaker original defect response. Second, feature fusion significantly improves the imaging results for both probes. By comparing Figure 20a with Figure 20c, and Figure 21a with Figure 21c, it can be seen that feature fusion enhances the defect visibility of the conventional probe by suppressing background noise and increasing image contrast. Similarly, comparison between Figure 20b,d, as well as between Figure 21b,d, shows that feature fusion further improves the results of the focused probe, leading to clearer defect morphology, more continuous boundaries, and stronger resistance to background noise. These results indicate that the fusion framework plays an important role in enhancing image contrast and robustness. Third, comparison between the aluminum-alloy–rubber and titanium-alloy–rubber results shows that image quality is strongly influenced by the original signal quality. In the titanium-alloy–rubber specimen, the non-fusion cases exhibit more pronounced background noise and less distinct defect boundaries, suggesting that weaker defect responses impose greater challenges on defect visualization. Although feature fusion improves the imaging performance in both material systems, its benefit is still constrained when the original defect response is too weak. This indicates that image-level enhancement alone is insufficient unless adequate signal quality is provided at the probe level.

Among the four configurations, the focused EMAT probe with feature fusion achieves the best overall performance in both specimens. This configuration provides the clearest defect morphology, the highest defect-to-background contrast, and the most complete boundary representation, thus offering the most reliable basis for subsequent defect segmentation and quantitative evaluation. Overall, the focused probe and the feature-fusion strategy play complementary roles: the former improves signal excitation and reception at the hardware level, whereas the latter enhances defect representation and noise suppression at the image-processing level.

#### 3.3.2. Performance Comparison Between Fusion Imaging and Single-Feature Imaging

To validate the effectiveness of the proposed multi-feature fusion framework, further comparisons were performed among three individual feature images, namely AMP, TDI, and PSD, as well as different fusion strategies, including equal-weight fusion, and entropy-weighted fusion.

**Figure 22:**
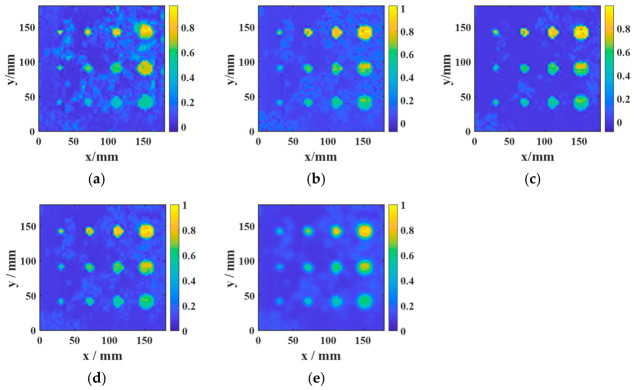
Comparison among AMP, TDI, PSD, equal-weight fusion, and entropy-weighted fusion for a representative aluminum-alloy–rubber specimen: (**a**) AMP; (**b**) TDI; (**c**) PSD; (**d**) equal-weight fusion; (**e**) entropy-weighted fusion.

**Figure 23:**
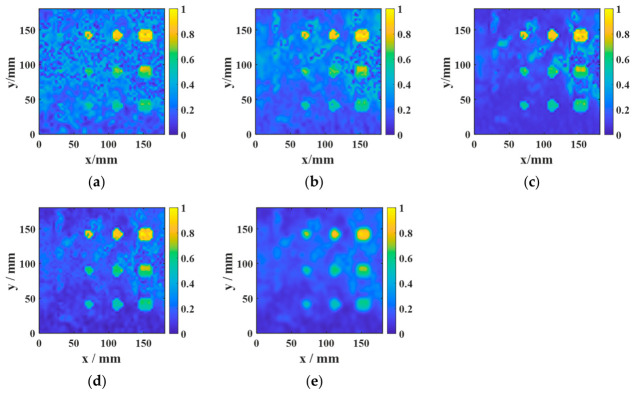
Comparison among AMP, TDI, PSD, equal-weight fusion, and entropy-weighted fusion for a representative titanium-alloy–rubber specimen: (**a**) AMP; (**b**) TDI; (**c**) PSD; (**d**) equal-weight fusion; (**e**) entropy-weighted fusion.

The AMP image directly reflects the variation in echo amplitude and is therefore sensitive to strong reflections from debonded regions. It can highlight defects with relatively strong responses, but it is also more susceptible to random noise, local spikes, and electromagnetic interference, which may result in elevated background artifacts and irregular defect contours. In contrast, the TDI image emphasizes the cumulative resonance energy in the time domain and generally exhibits better robustness to random fluctuations. However, the integration process tends to smooth the local response distribution, leading to broader and less distinct defect boundaries. The PSD image characterizes the concentration of resonance energy in the frequency domain and is particularly effective in distinguishing defect-related resonance components from broadband noise. As shown in Figure 22 and Figure 23, PSD-based imaging provides relatively stable defect visibility and better noise suppression under low-SNR conditions, although some spatial details may still be lost when it is used alone.

These results indicate that AMP, TDI, and PSD describe different aspects of the defect response and thus exhibit complementary advantages. Specifically, AMP offers high local sensitivity, TDI improves time-domain robustness, and PSD enhances frequency-domain discriminability. Therefore, no single feature can simultaneously achieve optimal defect contrast, boundary integrity, and noise robustness across different specimens and material systems.

The comparison between the two fusion strategies further confirms this point. Equal-weight fusion improves the overall image quality by combining the complementary information of the three feature maps, but its performance is limited by the fixed weighting scheme. By contrast, entropy-weighted fusion adaptively assigns larger weights to features containing more discriminative information and smaller weights to those more strongly affected by noise. Consequently, as shown in Figure 22 and Figure 23, entropy-weighted fusion yields the clearest defect representation and the most homogeneous background among all the compared methods, demonstrating superior effectiveness for defect visualization and subsequent quantitative evaluation.

The quantitative results in Table 7 and Table 8 are consistent with the visual observations in Figure 22 and Figure 23 and further demonstrate the advantage of feature fusion over single-feature imaging. For the aluminum-alloy–rubber specimen, all fusion-based strategies outperform the individual AMP, TDI, and PSD images, with entropy-weighted fusion achieving the highest SNR (14.269 dB) and contrast (0.7973). Compared with the best single-feature result (PSD), entropy-weighted fusion improves the SNR by 1.713 dB and the contrast by 0.1917, while still providing measurable gains over equal-weight fusion. A similar but more pronounced trend is observed for the titanium-alloy–rubber specimen, where entropy-weighted fusion yields the highest SNR (15.216 dB) and contrast (0.8745), exceeding equal-weight fusion by 0.947 dB in SNR and 0.0770 in contrast. These results confirm that AMP, TDI, and PSD capture complementary aspects of the defect response, whereas no single feature alone can simultaneously provide optimal defect enhancement, background suppression, and noise robustness. By adaptively weighting the three feature maps according to their discriminative information content, entropy-weighted fusion achieves more effective feature integration and produces the most discriminative defect representation. This advantage is particularly evident in the titanium-alloy–rubber specimen, where the original response is weaker and the imaging quality is more sensitive to noise contamination and feature selection.

Therefore, among the compared methods and for the representative cases studied here, entropy-weighted fusion provides the best overall performance in terms of SNR and contrast. This conclusion should be understood within the scope of the current experimental dataset rather than as a universal ranking for all possible inspection scenarios. Nevertheless, the present results suggest that adaptive fusion is a practical and effective strategy for improving defect representation when weak-response and noise-sensitive conditions are involved. In the present study, the quantitative comparison is based on representative cases acquired under identical experimental conditions.

## 4. Conclusions

This study proposed a non-contact framework for detecting debonding defects in metal–rubber bonded structures by combining a magnetic-field-enhanced focusing EMAT with entropy-weighted multi-feature fusion imaging. The results show that the proposed Halbach-type focusing magnet significantly enhances the vertical bias magnetic field, thereby improving Lorentz-force-driven ultrasonic excitation and strengthening defect-related responses. This provides a better basis for the detection of small debonding defects.

The comparative results further indicate that AMP, TDI, and PSD describe complementary aspects of the defect response, while entropy-weighted fusion achieves higher SNR and contrast than single-feature imaging and equal-weight fusion under the present experimental conditions. The ablation study shows that the focused probe and the fusion strategy play different but complementary roles; the former mainly enhances signal generation and defect-response amplitude at the hardware level, whereas the latter mainly improves image contrast, background suppression, and segmentation consistency at the image level.

Under the present experimental conditions, the proposed framework enables reliable detection of 5 mm debonding defects in aluminum-alloy–rubber specimens and 10 mm defects in titanium-alloy–rubber specimens. Overall, the results suggest that the combined use of magnetic-field focusing and adaptive multi-feature fusion is a promising approach for the detection and quantitative characterization of micro-debonding defects in metal–rubber bonded structures.

## Figures and Tables

**Table 1:** Experimental parameters.

Parameter	Excitation Frequency	Coil Diameter	Scan Step Size	Scan Range
Value	1.248 MHz	Ø10 mm	0.1 mm	180 mm × 180 mm

**Table 2:** Parameters of the focusing magnet.

Component	Layer	Outer Diameter (mm)	Inner Diameter (mm)	Remarks
Axially magnetized cylindrical magnet	Top	22	--	Cylinder; axial magnetization
	Middle	17	--	Cylinder; axial magnetization
	Bottom	12	--	Cylinder; axial magnetization
Axially magnetized annular magnet	Top	30	22	Ring; axial magnetization
	Middle	30	21	Ring; axial magnetization
	Bottom	30	15	Ring; axial magnetization
Transversely magnetized sector magnets (gap-filling)	Top	21	17	Multiple sectors form a ring; sector central angle 90–180°
	Bottom	16	12	Multiple sectors form a ring; sector central angle 90–180°
Layer height	Top/Middle/Bottom	15	15	15 mm

**Table 3:** Quantitative sizing results for the conventional probe in aluminum-alloy–rubber.

Thickness/Diameter	5 mm	10 mm	15 mm	20 mm
5 mm	--	11.86 (18.6%)	17.43 (16.2%)	22.34 (11.7%)
10 mm	--	11.42 (14.2%)	16.42 (9.4%)	21.15 (5.75%)
20 mm	--	10.68 (6.8%)	14.18 (5.4%)	22.36 (11.8%)

**Table 4:** Quantitative sizing results for the focused probe in aluminum-alloy–rubber.

Thickness/Diameter	5 mm	10 mm	15 mm	20 mm
5 mm	4.86 (2.8%)	10.92 (9.2%)	16.23 (8.2%)	21.91 (9.5%)
10 mm	5.51 (10.2%)	10.98 (9.8%)	16.38 (9.2%)	21.39 (6.9%)
20 mm	5.38 (7.6%)	10.62 (6.2%)	15.25 (1.7%)	20.85 (4.3%)

**Table 5:** Quantitative sizing results for the conventional probe in titanium-alloy–rubber.

Thickness/Diameter	5 mm	10 mm	15 mm	20 mm
5 mm	--	--	13.28 (11.4%)	13.94 (30.3%)
10 mm	--	--	14.4 (4.0%)	23.58 (17.9%)
20 mm	--	--	--	21.36 (6.8%)

**Table 6:** Quantitative sizing results for the focused probe in titanium-alloy–rubber.

Thickness/Diameter	5 mm	10 mm	15 mm	20 mm
5 mm	--	9.82 (1.8%)	13.78 (8.1%)	19.18 (0.9%)
10 mm	--	10.84 (8.4%)	16.51 (10.1%)	21.32 (6.6%)
20 mm	--	11.14 (11.4%)	15.88 (5.7%)	21.75 (8.7%)

**Table 7:** Quantitative comparison of SNR and contrast in the aluminum-alloy–rubber specimen.

Parameter	AMP	TDI	PSD	Equal-Weight Fusion	Entropy-Weighted Fusion
SNR (dB)	10.332	10.682	12.556	13.889	14.269
Contrast	0.5268	0.5723	0.6056	0.6778	0.7973

**Table 8:** Quantitative comparison of SNR and contrast in the titanium-alloy–rubber specimen.

Parameter	AMP	TDI	PSD	Equal-Weight Fusion	Entropy-Weighted Fusion
SNR (dB)	11.714	13.081	13.889	14.269	15.216
Contrast	0.6848	0.7784	0.7815	0.7975	0.8745

## Data Availability

The data presented in this study are available on request from the corresponding author after obtaining permission of an authorized person.

## Abbreviations

The following abbreviations are used in this manuscript:

EMAT	Electromagnetic Acoustic Testing
NDT	Nondestructive Testing
SNR	Signal-to-Noise Ratio
PSD	Power Spectral Density
EMUR	Electromagnetic Ultrasonic Resonance
DFT	Discrete Fourier Transform
